# Developmental implications of prenatal opioid exposure among school-aged children: a mixed methods and community-initiated analysis

**DOI:** 10.1186/s12889-023-16702-8

**Published:** 2023-09-19

**Authors:** Andra Wilkinson, H. S. Rackers, T. M. Harmon

**Affiliations:** 1 Child Trends, Inc, 1516 E Franklin St, Chapel Hill, NC 27514 USA; 2 To The Moon And Back, PO Box 1078, Plymouth, MA 02362 USA

**Keywords:** Neonatal opioid withdrawal syndrome, Prenatal opioid exposure, Child development, Mixed methods, Community collaboration

## Abstract

**Background:**

Growing research points to potential long-term developmental implications of prenatal opioid exposure for children. Yet, polysubstance use and adverse childhood experiences are raised as potential confounders. Further, there is a lack of data on school-age children and the children’s strengths.

**Methods:**

Parents and caregivers of children with prenatal opioid exposure worked with the study team to design, collect, and descriptively analyze mixed method data. Data were collected through survey (*n* = 148) and two focus groups (*n* = 15) from a convenience sample in mostly West Virginia and Massachusetts.

**Results:**

Nearly half of the children in the sample were diagnosed with multiple developmental delays, behavioral health conditions, and specific learning disorders. Roughly 85% of children have behavioral challenges. Associations between prenatal opioid exposure and negative developmental outcomes did not vary by type of opioid nor by polysubstance use, while controlling for adverse childhood experiences. Importantly, over 80% of families also reported their child’s strengths, including empathy, social magnetism, and their resilience.

**Conclusions:**

The challenges for children born with prenatal opioid exposure may extend into school-age. The results are consistent with prior research on younger children, suggesting a need for best practices for caring for these children beyond the neonatal stage.

## Significance

*“What is already known on this subject?*

*Prenatal opioid exposure is associated with cognitive, psychomotor, language, and behavioral implications in development for infants, toddlers, and preschoolers. Though potential confounders (e.g., polysubstance exposure, trauma) may complicate the relationship.*

*“What this study adds?*

*The developmental implications of prenatal opioid exposure appear to extend into school age and do not appear to vary by type of opioid or by polysubstance use. Adjusting for trauma did not change the association between prenatal opioid exposure and negative developmental outcomes.*

## Background

From 2004 to 2014, the incidence of neonatal abstinence syndrome (NAS) increased more than fivefold among births covered by Medicaid [1]. NAS is a group of conditions that babies can experience when withdrawing from certain substances in the womb, especially opioids [2]. NAS can be characterized by irritability, difficulty feeding, difficulty sleeping, and seizures in infants [2]. Emerging research suggests children with prenatal opioid exposure (POE) can experience a range of longer-term developmental and behavioral challenges.

Four meta-analyses and a systematic review point to cognitive, motor, language, and behavioral deficits among children with POE in mainly the toddler and preschool stages of development. Yeoh et al., 2019 examined 26 studies comparing prenatal exposure to any opioid including heroin, methadone, buprenorphine, and polysubstance use to drug-free controls and found significantly lower cognitive scores in the toddler and preschool age ranges as well as significantly lower motor scores for children with POE [3]. Monnelly et al., 2019, focusing only on prenatal methadone exposure (including polysubstance exposure), found very similar outcomes with the Mental Development and Psychomotor Development Indices being significantly lower at two years of age in children with prenatal methadone exposure compared to children without [4]. Across the 41 included studies, seven of the studies also examined behavioral scores and in six of the seven, negative behavioral outcomes were reported among children with prenatal methadone exposure.

Lee et al., 2020 extended these prior studies by being able to examine development on a wider range of measures [5]. Across 16 studies, defining POE to include any opioids including methadone and buprenorphine, this analysis found infants and young children with POE had significantly lower cognitive, psychomotor, and language scores with also more parent-rated internalizing, externalizing, and attention problems. Focusing specifically on the attention problems and among older children (ages 4–11 years), Schwartz et al., 2021 in a review of seven articles, found POE (including polysubstance exposure) was positively associated with hyperactivity/impulsivity, inattention, and combined ADHD symptoms scores in both preschool and school-age children [6]. Most recently, Balalian et al., 2023 examined 79 studies and found POE was associated with worse cognitive and motor development in children [7]. However, this review could not do a meta-analysis given the considerable heterogeneity among the studies in the outcomes examined, the types of opioids and other prenatal exposures included and during which period of the pregnancy, among other factors.

Additional limitations of the current literature is the lack of detailed data on potential confounders. Yeoh et al., 2019, Monnelly et al., 2019, and Lee et al., 2020 all note that few studies controlled for potential confounders including socioeconomic status or environmental factors [3–5]. Lee et al., 2020 further notes that the social determinants of health associated with opioid use disorder in pregnancy are also potential confounders of the associations between POE and child outcomes [5]. Given the high prevalence of child welfare involvement among children with POE [8], adverse childhood experiences are also a potential confounder in the association between POE and developmental outcomes [9].

The present study aims to address several of the limitations in existing literature by collecting data from school-age children on: (1) polysubstance exposure, including the type of substance and at what point in the pregnancy; (2) adverse childhood experiences as an important potential confounder; and (3) a wide range of developmental and behavioral outcomes, including positive ones. Finally, this study was co-designed and co-led with parents raising children with POE both because, unquestionably, they know these children best, and because we wanted the results to be meaningful for the families raising these children.

## Methods

This study was community-initiated. To The Moon And Back is a nonprofit serving children and families with POE since 2017 in Massachusetts and West Virginia. To The Moon And Back contacted Child Trends to request we collect data from the families they are serving. We used a mixed methods approach, specifically a convergent parallel mixed-methods design, to give equal priority to the quantitative and qualitative data, and to avoid one informing or constraining the other [10]. We collected survey and focus group data from and in collaboration with families in West Virginia and Massachusetts. The research questions included the following, the results for the third research questions are not reported in this paper:

1. What are the developmental implications of prenatal opioid exposure, both positive and negative?
2. Do the developmental outcomes associated with prenatal opioid exposure vary by type of opioid or by polysubstance use, while controlling for adverse childhood experiences?
3. What services do families of children with POE use, which are helpful, and what services do they wish existed?

The survey provided mostly quantitative and some qualitative data from a larger sample that made it feasible to understand the prevalence and range of developmental implications of POE (research question 1) and service utilization and experiences (research question 3). The quantitative data from the survey also made it feasible to test whether developmental outcomes differed by type of opioid exposures (research question 2). The focus groups with families provided qualitative data that added considerable depth to our understanding of not only developmental implications (research question 1) and service utilization (research question 3) but how families understand, describe, and navigate them. The convergent parallel mixed-method design finally allowed for an analysis of whether the findings from the quantitative and qualitative results converge or diverge [10].

### Sample

Child Trends partnered with To The Moon And Back in all parts of this research to ensure those with lived experience informed the research questions, the mode of data collection, and data analysis. For example, we learned families found the new term for NAS, NOWS, confusing, so we used NAS in this paper. Child Trends had no relationship with the participating families outside of To The Moon And Back.

### Survey sampling and analysis

Researchers worked with To The Moon And Back to develop the survey, test the questions, and distribute the survey to a convenience sample via their website and partners in West Virginia and Massachusetts. To The Moon And Back distributed the survey through their listserv, their social media platforms, and through their partner organizations, including Early Intervention, mental health providers, foster and adoptive support groups, and support groups for parents in recovery. Caregivers of children with POE in any state were eligible to participate, and the survey was self-administered. Survey domains included: prenatal substance exposures (type, timing, frequency), post-delivery infant care, child welfare involvement, adverse childhood experiences, clinical diagnoses, challenging behaviors, strengths, and measures of health and education service utilization across roughly 65 questions. The present study does not include the survey results around service utilization. Surveys were collected and managed using the secure, web-based Research Electronic Data Capture (REDCap) tools hosted at Child Trends [11, 12]. The survey was open from February through May of 2022. Two hundred and fifteen surveys were collected, and 67 were dropped from the analytic sample due to being ineligible as they were not raising a child with POE or the surveys were incomplete, leaving 148 surveys for the analytical sample. Descriptive and statistical analyses were conducted in STATA. T-tests were conducted to determine if there was a significant difference in the mean child age between those who positively endorsed an outcome and those who did not. Logistic and ordered logistic regressions were conducted to test for relationships between outcomes of interest and opioid-only exposure and polysubstance exposure, as well as between those who were exposed to prescribed opioids and those who were exposed to only illicit opioids. The regression models controlled for ACEs exposure, age, state, and race/ethnicity. Open-text survey responses were analyzed using frequentist and inductive qualitative data analysis techniques. Open-text survey responses were analyzed using frequentist and inductive qualitative data analysis techniques.

### Focus group sampling and analysis

Researchers collaborated with To The Moon And Back to develop the focus group protocol [13], design the sampling frame, and recruit participants. Families were recruited for the focus groups through the same channels and partnerships through which the survey was distributed. Participants were recruited through purposive, convenience, and snowball sampling to try and ensure participation by a mix of foster and birth families raising children with POE. Participants were recruited primarily online or through face-to-face conversations between To The Moon And Back staff or partners and parents and caregivers. Focus groups were co-facilitated by one of the paper authors with prior focus group facilitation training and experience (Wilkinson, Ph.D., female) and a parent raising children with POE to maximize the comfort of the participants in the group. The co-facilitator had relationships with some of the participants prior to the study, the author facilitator had no relationships with the participants prior to the study. The participants were informed of the goals for the study prior to the focus groups beginning. The methodological orientation for the focus groups was content analysis. The focus group protocol had five questions broadly assessing: 1) positive and negative experiences raising children with POE; 2) which services the children are using; 3) which services families found helpful services; 4) which services they wish existed; and 5) what they wish providers knew about their child. The present study includes the results from the first focus group question. Two participants were unable to attend the focus group due to child care disruptions and illness. The focus groups were conducted via Microsoft Teams, a video conference platform, and lasted 60–90 min. The focus groups were conducted in the evening so participants were primarily at home. Field notes were taken during and after the interview, during a debrief between the facilitators. Two focus groups, one in Massachusetts (9 participants) and one in West Virginia (6 participants), had a total of 15 participants. Focus groups were automatically transcribed and video recorded. Transcriptions and recordings were analyzed using frequentist and inductive methods with a priori codes based on the protocol and probes and inductive codes as the analysis progressed. One coder coded the data, and the results were checked by a participant. There were minimal code variations between the focus groups, an indication of saturation.

### Ethics statement

This research was conducted following the protocol reviewed and approved by the Child Trends Institutional Review Board. The survey took an estimated 15–20 min to complete, and participants were entered into a raffle for a $50 gift card for their time. Focus group participants received a $15 Amazon gift card as an incentive.

## Results

### Sample characteristics

Most survey respondents were from Massachusetts (64%), followed by West Virginia (11%), with an additional 21 states represented (Table 1). Caregivers were primarily White Non-Hispanic (93%), with undergraduate or graduate degrees (48%). Children had a mean age of 6 years old (range from 0 to 24 years, SD: 4.59), roughly a quarter were multi-racial, and they commonly resided with adoptive parents (66%), relatives (15%), or non-relative foster parents (14%). Birth parents were eligible but represented only 4% of the sample. Families with multiple eligible children were asked to complete a survey for each child. In the focus groups, the average child’s age was seven years old, and the caregivers were also primarily adoptive and foster parents.

**Table 1:** Sample Characteristics, *n* = 148

	Freq	%
State, *n* = 148		
Massachusetts	94	64%
West Virginia	17	11%
Not listed	37	25%
Current Caregiver, *n* = 146		
Adoptive parent(s)	98	66%
Relative(s)	22	15%
Non-Relative foster parent(s)	20	14%
Biological parent(s)	6	4%
Missing	2	1%
Caregiver Education Level, *n* = 148		
High School or GED	12	8%
Some College	25	17%
Certification, Vocational or Technical School Training	16	11%
Associate Degree	18	12%
Undergraduate Degree	35	24%
Graduate Degree	42	28%
Caregiver Race/Ethnicity, *n* = 148^a^		
White Non-Hispanic	138	93%
Hispanic	5	3%
Child Race/Ethnicity, *n* = 138^a^		
White Non-Hispanic	103	70%
Multiracial Non-Hispanic	34	23%
Black Non-Hispanic	7	5%
	Mean	SD
Caregiver Age, *n* = 148	44.84	9.25
Child Age, *n* = 147	5.65	4.59

^a^Totals do not add to 100%. Additional race/ethnicity categories were suppressed due to small sample size

### Substance exposures

The types of opioids children had prenatal exposure to included illicit opioids (74%), methadone or buprenorphine (43%), non-prescribed opioids (34%), and prescribed opioids (11%) (Table 2). The majority of the sample (85%) were exposed to additional substances prenatally, and on average they were exposed to four additional substances. The additional substances included nicotine (94%), marijuana (89%), alcohol (76%), tranquilizers (71%), methamphetamines (66%), other stimulants (61%), and hallucinogens (24%). When asked about which trimester in the pregnancy the child was exposed, the most common answer was throughout the pregnancy, only a few respondents reported first and second trimester exposures only for alcohol, nicotine, and/or marijuana. Rates of polysubstance use did not vary between pregnant people using prescribed opioids, methadone, or buprenorphine and pregnant people using non-prescribed or illicit opioids. Further, exposure to illicit opioids was common for the majority of pregnant people using methadone or buprenorphine. Logistic and ordered logistic regressions did not produce significant findings when comparing developmental outcomes between pregnant people with polysubstance exposure to pregnant people with opioid-only exposure as well as those with opioid treatment (prescribed opioids and/or medications for opioid use disorder) compared to those without illicit-only exposure. These models controlled for exposures to ACEs and this term was not statistically significant in any of the models.

**Table 2:** Prenatal Exposures, *n* = 148

	Freq	%
POEs		
Illicit Opioids	110	74%
Medication Assisted Treatment	64	43%
Non-prescribed opioids	50	34%
Prescribed opioids	17	11%
Don't know	16	11%
Prenatal Exposure to Other Substances		
Yes	126	85%
No	-	< 5%
Don’t know	21	14%
Other Substance Exposures		
Nicotine/cigarettes, *n* = 100	94	94%
Marijuana, *n* = 83	74	89%
Alcohol, *n* = 71	54	76%
Methamphetamine, *n* = 71	47	66%
Tranquilizers or sedatives, *n* = 52	37	71%
Other stimulants, *n* = 51	31	61%
Hallucinogens, *n* = 34	8	24%
	Mean	SD
Mean number of substances exposed, *n* = 106	4.12	1.47

### Neonatal treatment

Characteristics of the survey sample’s neonatal treatment suggest considerable severity of cases (Table 3). In the study sample, 80% of children were diagnosed with NAS. In addition, 74% of children were hospitalized for more than two weeks, and at least 68% received pharmacological treatment, morphine being the most common. Only 5% of infants roomed in with family, while 89% spent most of their time in specialty care, the neonatal intensive care unit or a specialty care nursery being the most common.

**Table 3:** Neonatal treatment, *n* = 148

	Freq	%
Diagnosed with Neonatal Abstinence Syndrome (NAS)		
Yes	119	80%
No	15	10%
Don't know	14	9%
Where Child Spent Most of Hospital Time, *n* = 119		
Neonatal intensive care unit (NICU)	80	67%
Special Care Nursery	26	22%
In the room with family	6	5%
Don’t know	7	6%
Length of Hospital Stay, *n* = 119		
Less than 2 weeks	25	21%
Between 2 and 3 weeks	29	24%
Between 3 weeks and a month	32	27%
More than a month	27	23%
Don’t know	6	5%
Medication to Treat NAS Symptoms, *n* = 119		
None	16	13%
Morphine	64	54%
Methadone	18	15%
Phenobarbital	18	15%
Don't know	23	19%

### Adverse childhood experiences

Exposures to adverse childhood experiences (ACEs) were reported by 50% of the sample (Table 4). Interestingly, 70% of the children in the sample had experienced a removal from their birth family. The median number of removals was one and the most common age of removal was under the age of one. The most common ACES reported were residing with an adult who used alcohol or drugs (39%), residing with an adult who had a mental illness or attempted suicide (30%), parents being separated or divorced (28%), and residing with an adult who was sentenced to and/or served time in the justice system (25%). T-tests revealed older children were more likely to have experienced an ACE, for all the ACEs.

**Table 4:** Adverse Childhood Experiences

	Freq	%
Adverse childhood experiences, *n* = 142^a^		
No adverse experiences	62	44%
Resided with adult who used alcohol or drugs	56	39%
Resided with adult who had mental illness or attempted suicide	43	30%
Parents separated or divorced	40	28%
Resided with adult who was sentence to/served time in prison or jail	35	25%
Physical abuse	27	19%
Verbal or emotional abuse	24	17%
Don't know	8	6%
Ever removed from the home, *n* = 146	102	70%
	Mean	SD
Mean number of adverse experiences, *n* = 140	1.63	2.01
Mean number of removals, *n* = 102	1.33	0.65

^a^Sexual abuse ACE is suppressed due to small sample size

### Diagnoses and delays

The range of diagnoses demonstrates the complexity of issues amongst survey respondents (Table 5). Participants mainly reported diagnoses of developmental delays (63%, 72% reporting more than one), behavioral health conditions (45%), and specific learning issues (28%). Forty-nine percent reported multiple diagnoses, primarily across developmental delays, behavioral health conditions, and specific learning disorders. The most common diagnosed developmental delays reported included language (71%), physical (59%), social and emotional (59%), and cognitive (49%). Focus group participants provided examples of their children’s delays: unable to sit at 16 months, nonverbal at four years old, unable to draw a straight line at four years old, and testing two grades behind. Parents in the focus groups reported additional diagnoses, including gastrointestinal issues, ADHD, and Autism. T-tests revealed older children were more likely to be diagnosed with neurological disabilities, learning disabilities, and behavioral health conditions.

**Table 5:** Developmental Diagnoses and Delays

	Freq	%
Developmental Diagnoses, *n* = 142		
None	26	18%
Developmental delay	89	63%
Behavioral health condition	64	45%
Specific Learning Issues	40	28%
Physical disability	26	18%
Neurological disability	14	10%
Intellectual disability	-	< 5%
Don't know	-	< 5%
Multiple developmental diagnoses, *n* = 142	70	49%
Developmental Delays, *n* = 86		
Language	61	71%
Physical	51	59%
Social and emotion	50	58%
Cognitive	42	49%
Don't know	-	< 5%
Multiple development delays, *n* = 86	62	72%
	Mean	SD
Number of developmental diagnoses, *n* = 142	1.65	1.35
Number of developmental delays, *n* = 86	2.37	1.14

Several factors complicated how parents in the focus groups understood their children’s challenges. Parents remarked how the challenges varied between children with similar exposures. Parents were unsure which challenges their children would outgrow and which warranted intervention. Parents were also wary of diagnoses given to their children because they did not think they were appropriate or were not needed. Examples include *“…Everybody wants to throw a diagnosis at my baby.”* As well as *“I know that they have to be diagnosed, but…I don't want him to have the ADD diagnosis 'cause I don't really believe that is his issue. I don't want him labeled, and I don't want him mislabeled*.”

In the focus groups, several parents mentioned the challenging social implications for themselves and their children: *“I’ve lost friends”* and *“most people can’t relate.”*

*“first thing, I always think of is someone going to call and say we, we can't have yours in school anymore. Like, it's so scary. But I don't want to not have my kids participate in all the other enrichments and things that other kids participate in just because they're a little bit more difficult at times.”*

This issue extended to concerns over their child’s right to privacy. Parents were frustrated that they must explain their kid’s background to each new provider in front of their kid, who may be old enough to start understanding.

*“…They don't yet have the skills to be able to manage well on their own. And I know that, but I don't think I should have to explain the basis and origin of it to everyone because my kids should have privacy too.”*

### Behavioral issues and tantrums

Results from behavioral questions on the survey included in Table 6 provide insight into the complexity of the behavioral and symptom profile of participating children. While the questions about development focused on diagnoses only, the questions about behavior reflect parent and caregiver observations and opinions. Over half of respondents endorsed impulsivity (67%), tantrums (60%), difficulty with transitions (58%), aggression (57%), seeking or avoiding sensory input (57%), and difficulty with changes in routine (55%). Of those reporting tantrums, nearly half (45%) report an increase in tantrums over the past year. Of those reporting sensory processing issues, children varied for each sensory input on whether they avoid it or seek it. For example, of the children who have sensory processing issues with light, 60% avoid light and 40% seek it. There were more clear patterns of sensory avoidance for sound and clothing and clearer pattens of sensory seeking for physical touch. For smells and tastes, equal numbers of children were reported to seek and avoid them. The focus group participants showed how these behavioral issues could manifest. They said their children would bounce while sitting or bang their heads against walls to get physical sensory input. Regarding impulsivity, one parent said, *“I’m on like speed dial with poison control. They know me by my first name.”* Parents also said their children can struggle to self-regulate, *“He goes from zero to 100 very fast…from even infancy.”*

**Table 6:** Behavioral Issues and Tantrums

			Freq	%
Behavioral Issues, *n* = 141				
None			11	8%
Impulsivity			94	67%
Tantrums			84	60%
Difficulty with transitions			82	58%
Aggression			80	57%
Seeking or avoiding sensory input			80	57%
Difficulty with changes in routine			78	55%
Forgetful			59	42%
Slow to respond to cues			52	37%
Low body awareness			46	33%
Don't know			-	< 5%
Tantrum Length, *n* = 84				
About 30 min or less			48	57%
About 30 to 60 min			30	36%
1—2 h			-	< 5%
2 h or more			-	< 5%
Past Year Increase in Tantrums, *n* = 84				
No			43	51%
Yes			38	45%
Don’t know			-	< 5%
			Mean	SD
Mean number of tantrums, *n* = 82			3.24	0.98
	Avoids		Seeks	
Sensory Experience	Freq	%	Freq	%
Light	27	60%	18	40%
Sound	46	70%	20	30%
Physical touch	13	21%	50	79%
Clothing	38	78%	11	22%
Smell	23	50%	23	50%
Taste	27	49%	28	51%

In open-ended survey items, parents reported atypical triggers for their child’s tantrums, including overstimulation (e.g., baths, haircuts, loud noises) and when their child cannot complete a task. Other parents connected tantrum triggers to experiences the child may have had. As one parent noted, "[*Child's] tantrums are always related to fear of his basic needs not being met, abandonment, and fear. He is displaying PTSD from incident during reunification."* Nearly one-third of parents also reported tantrums could be aggressive or violent, including pushing, hitting, spitting, biting, kicking, head banging, hair pulling, breaking toys, and throwing things: *"bedroom door has huge dents in it"* and *"gave me bruises more than once."* More than 10% reported that tantrums have an intense or unpredictable quality: *"She can go from laughing and happy to extremely angry in seconds without warning”* and *"There is no reasoning when tantrums start. You just have to make sure everyone is safe."*

### Strengths

We asked parents to describe their children’s strengths in both the surveys and focus groups. In the survey, 122 parents reported on their child’s strengths, and 39% said intelligence (*“He's so smart. That is one of the joys these kids are so smart. It…just, like, amazes me.”*), another 39% said empathy (*“He's the kindest person I've ever met. Like, I don't even know how kind he is. He just cares so much.”*), and 31% praised their child’s social skills (*"Most people are drawn to him"* and* “[Child] is a bright shining star. He excels at social interaction. Loves to meet new people.”*).

In the focus groups, the parents were particularly proud of their child’s resilience: *“We always say what these kids can't do, but we've never. We haven't figured out yet what they can do,” “Sometimes what we wanna like just run out of the room with is their resilience and it is what they have used to survive,” “Every little piece of progress. It's just so amazing to see because she works so hard for everything.”*

### Convergence

In alignment with a convergent parallel mixed method design, the final step of analysis is to examine whether the survey and focus group data converge [10]. The study collected both qualitative and quantitative data on three domains: developmental diagnoses, behavioral issues, and strengths. Both the qualitative and quantitative data point to a wide and significantly overlapping range of developmental diagnoses among children with POE. The qualitative data extended the results by revealing that some parents are concerned their children with POE are often misdiagnosed and that the diagnoses can have social implications for the children and their families. For behavioral issues, the survey and focus group data converged most for tantrums and impulsivity, the issues most endorsed by the survey respondents. For the issues slightly less commonly endorsed by the survey respondents (e.g., sensory processing issues), there was thinner convergence, with a few focus group participants mentioning the issues. Where there was convergence on behavioral issues the qualitative data added detailed examples that deepened understanding of the behavioral issues and how they could impact family life. For strengths, there was little convergence between the survey and focus group. The focus group participants really centered on resilience when discussing their children’s strengths and the survey participants mentioned intelligence, empathy, and social magnetism. A potential explanation for the lack of convergence on strengths is parents may find it easier to enumerate their children’s strengths in the anonymity of the survey than in a focus group with other parents and caregivers.

## Discussion

The present study collected quantitative and qualitative data from and in partnership with parents and caregivers raising children with POE. Parents shared the challenges and joys of raising these children and provided details on the children’s exposures. This study extends the existing literature in several important ways. First, where the bulk of existing research on the developmental implications of POE is focused on infants, toddlers, and preschoolers [3–5], the present study collected data on school-age children. Second, existing literature notes the prevalence of polysubstance exposure among children with POE [3–5]. The present study provided information on the breadth of substances used and when during the pregnancy. Further, this study tested if outcomes varied between children with polysubstance exposure compared to children with just opioid exposure. Third, the present study extends exposures from substances to be inclusive of adverse childhood experiences, controlling for these in statistical models. Finally, in an attempt to examine the implications of POE for the whole child, and not just their reading scores, the present study asked parents and caregivers about their children’s strengths as well as their struggles, and cast a wide net to capture a myriad of developmental implications.

Overall, the results extend our understanding of the cognitive, motor, language, and behavioral challenges children with POE may have into school-age. While roughly one in six (17.3%) U.S. children and adolescents aged 3–17 years will have developmental delays, disorders, and disabilities [14], 63% of our sample were diagnosed with a developmental delay. An estimated 1 in 5 children will have an identified mental health condition in a year in the U.S. [15], and 45% of our sample reported a behavioral health diagnosis. The results also point to the considerable complexity of the exposures the children experienced from an average of five different substances throughout pregnancy to adverse childhood experiences, including high levels of involvement with the child welfare system. Though polysubstance exposure and ACEs complicate the associations between opioid exposure and developmental outcomes, the present study found no variation in outcomes by types of opioid and poly substance exposure, while controlling for exposure to ACEs.

Future research should continue to explore the complexity of these associations; this study’s results were not statistically significant, but testing this among more representative samples is important. Further, the present study, in asking parents and caregivers to share their children’s strengths, raises additional questions for future research. For example, some parents and caregivers reported their children were incredibly smart, some had even been tested as gifted, while others reported significant cognitive delays. This complicates the potential argument that POE is associated with cognitive impairment. As another example, the prevalence of social and emotional delays and intense and even violent tantrums could lead to an assumption these children could struggle socially. And yet, some parents and caregivers were most proud of the social magnetism of their children and their ability to make fast friends. This suggests potential nuance and variation in the development of children with POE that should be explored.

The limitations of the present study include limited external validity as the sample is less representative than prior research, not only because it is smaller, Whiter, concentrated in two states, and mostly foster and adoptive parents, but also because it may be a more severe sample. This sample had a higher prevalence of polysubstance use [16] and longer lengths of stays in the hospital [17] compared to prior research. Perhaps a survey asking parents about the development of their child with POE may attract more parents with concerns. Another limitation is the use of parent and caregiver report. The survey was careful to ask about only diagnoses for developmental issues but did rely on parent and caregiver observations when asking about challenging behaviors. That the bulk of the parents were foster and adoptive parents limited our understanding of the polysubstance exposures. These caregivers likely had an imperfect understanding from the birth parent (e.g., for relative caregivers) and the child welfare system and there were high rates of respondents answering “don’t know” for these questions. Future research should center the perspectives of birth parents. While the survey sample reported the children had relatively few ACEs, birth parents may have more and different information. Finally, saturation of the qualitative data would have been stronger with additional focus groups.

In conclusion, with prenatal opioid use continuing to rise [18] and research indicating significant developmental implications of POE extending into school age, states must invest in services for these children and their families while research continues to gain medical clarity. While there are best practices for how to care for neonates with POE, there are no best practices for taking care of the toddlers, preschoolers, and school-age children with POE, who can face significant challenges. Lacking best practices in how to support these children across their developmental trajectory, they are at risk of educational and medical care inequities. Parents told us their Kindergartners had been referred to juvenile justice programs for their behavior, their children had been diagnosed with oppositional defiance disorder, and child care providers had hit or mistreated their children in frustration. Rather than punishing these children for having challenges beyond their control, we should support them and their families and help these kind and resilient shining stars thrive. The more providers and teachers understand these children and how to support them, the better for all involved.

## Data Availability

Data from the focus groups cannot be de-identified sufficiently to protect respondent confidentiality and thus cannot be shared. Quantitative data from the surveys are available from the corresponding author on reasonable request and with permission of To The Moon And Back.
